# Analysis of Rattleback Chaotic Oscillations

**DOI:** 10.1155/2014/569386

**Published:** 2014-01-08

**Authors:** Michael Hanias, Stavros G. Stavrinides, Santo Banerjee

**Affiliations:** ^1^Department of Electronics, Computers, Telecommunications and Control, Faculty of Physics, National and Kapodistrian University of Athens, Athens, Greece; ^2^Electrical and Computer Engineering Department, University of Cyprus, Nicosia, Cyprus; ^3^Laboratory of Cryptography, Analysis & Structure, Institute for Mathematical Research, University Putra Malaysia, Malaysia

## Abstract

Rattleback is a canoe-shaped object, already known from ancient times, exhibiting a nontrivial rotational behaviour. Although its shape looks symmetric, its kinematic behaviour seems to be asymmetric. When spun in one direction it normally rotates, but when it is spun in the other direction it stops rotating and oscillates until it finally starts rotating in the other direction. It has already been reported that those oscillations demonstrate chaotic characteristics. In this paper, rattleback's chaotic dynamics are studied by applying Kane's model for different sets of (experimentally decided) parameters, which correspond to three different experimental prototypes made of wax, gypsum, and lead-solder. The emerging chaotic behaviour in all three cases has been studied and evaluated by the related time-series analysis and the calculation of the strange attractors' invariant parameters.

## 1. Introduction

Behaviour of dynamical systems is always of great interest, especially when these dynamics reveal a nonlinear-chaotic behavior. Rattleback is such a case and there have been numerous analyses of its peculiar behaviour, since the 1890's [1, 2]. Many other papers have been published on the issue, especially during the 1980's [3–5].

Rattleback is a canoe-shaped body, that is, a semiellipsoid object, known from the ancient years named as “celt” or “anagyre.” It demonstrates the very interesting property of spin asymmetry leading to a peculiar kinematic behaviour. This property seems to be unexpected in first sight, due to its symmetrical shape. The demonstrated behaviour consists of a reasonably smooth spin in one direction, while in the opposite direction it develops a pitching instability that leads to spin reversal, in an apparent defiance of the principle of conservation of angular momentum.

It is apparent that rattleback provides a prototype of chiral dynamics, where in lack of mirror-symmetry it leads to unconventional dynamics. The first mathematical model was introduced by Walker [6], who studied the linearized rattleback equations of motion and concluded that the completely stable motion is possible in only one (clockwise) spin direction. It has already been reported that rattleback is demonstrating a chaotic behaviour during the procedure of reversing its spin [7–9].

In this paper, this chaotic behaviour for different variables is studied and evaluated by utilizing a mathematical model, whose parameters have been experimentally defined by three different rattlebacks made of wax, gypsum, and solder. Time-series analysis and the corresponding chaotic evaluation reveal the global dynamical features of this interesting object dynamical behaviour. The paper is organized in three sections. In the first section, rattleback's dynamics are introduced, utilizing Kane's model [4]. It is shown, by means of numerical solutions of full, non-linear motion equations that one can construct a realistic mathematical model by assuming rolling without slipping and employing a torque proportional to the angular velocity in order to provide for energy dissipation. Rattlebacks made of different materials such as wax, gypsum, and lead-solder have been constructed and Kane's model parameter values were experimentally determined, in order to study their dynamical behaviour. In the second section, time-series presentation for three (of the six) variables appears. These time-series have been numerically calculated and they demonstrate irregular behaviour, hinting chaos. Finally in the third section, time-series analysis is performed according to Grassberger-Procaccia method [10]. Rattleback's strange attractors' invariant parameters as correlation and minimum embedding dimension are also calculated, in an effort to reveal and confirm its global dynamics.

## 2. Rattleback's Dynamical System Experimental Model

**Figure 1:**
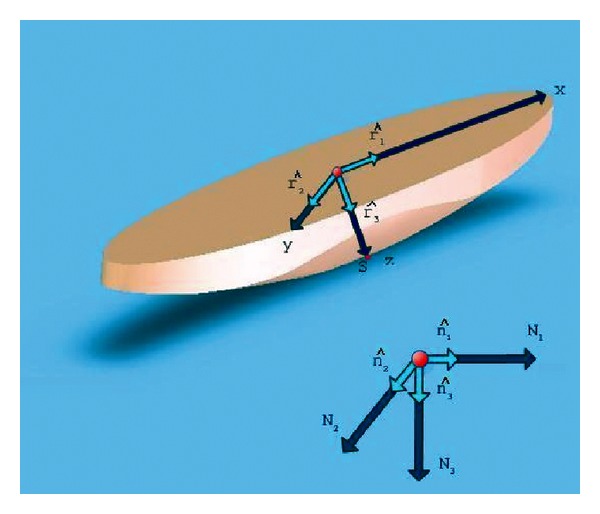
Rattleback's shape and its axes of coordinates.

Objects having the semielliptic, canoe-looking shape of rattleback, appearing in Figure 1, provide for a prototype of chiral dynamics, where the lack of mirror-symmetry leads to unconventional dynamics. In an attempt to summarize rattleback's curious mechanical behaviour one could say that this object, when spun on a flat horizontal surface in the clockwise direction, continues to spin in the same direction, until it consumes all its initial spin energy. But when it is spun in the counterclockwise direction, spinning soon ceases, the body briefly oscillates, and then reverses its spin direction in the clockwise direction, until all of its energy is again consumed.

The probing property of spin asymmetry, although unanticipated in a geometrically symmetrical object, it is apparent. Thus, while rattleback spins reasonably smoothly in one direction, it develops a pitching instability when it spins in the opposite direction leading to spin reversal, in an apparent defiance of the principle of conservation of angular momentum. But this is not the case.

The oscillations appearing just before rattleback reverses its spin direction, have already been reported in [7–9] that they are chaotic and are provided with deterministic chaotic features.

As already mentioned, the first mathematical model having the ability to represent these phenomena was proposed by Walker [6] in 1979 and it incorporated a linearized approach of rattleback's dynamics, proving that only one spinning direction could be stable (clockwise). Later, in 1982 Kane and Levinson proposed another very realistic mathematical approach [4] based on a set of six nonlinear ordinary differential equations (NODE) that are presented in (1).

Consider

(1) dadt=ω3sinβ+ω1cosβ,dβdt=(−ω3cosβ+ω1sinβ)tanα+ω2,dγdt=(ω3cosβ−ω1sinβ)sec α,dω1dt=E1G,dω2dt=E2G,dω3dt=E3G.

The first three variables *α*, *β*, define *γ* and the ellipsoid orientation; *α* stands for the roll angle, *β* for the pitch angle, and *γ* for the yaw angle. The other three variables *ω*_1_, *ω*_2_, and *ω*_3_ are the ellipsoid's spin rates, that is, the corresponding angular velocities. *G* is the determinant for mass of inertia, while *E*_1_, *E*_2_, and *E*_3_ are the determinants for the angular accelerations. These determinants are defined by (2), (5), (6), and (7), respectively, according to the following:

(2) G  =|A+m[x22+(h−x3)2]D−m·x1·x2m·(h−x3)·x1D−m·x1·x2B+m[x12+(h−x3)2]m·(h−x2)·x1m·(h−x3)·x1m·(h−x2)·x1C+m·(x12+x22)|.

*A*, *B*, *C*, *D* are the moments of inertia with respect to axes whose origin is at the center of mass and which are rotated by an angle *d* = 0.5 degrees [4]. It is important to note that the center of mass is at a position defined as *h* = (3/8)*c* and the axes whose origin is at center of mass are parallel to the geometrical axes of symmetry after a rotation by *γ*, *α*, *β*. The *A*, *B*, *C*, *D* are defined by the following set of four equations:

(3) A=(Ixx·cos2d+Iyy·sin2d)−m·h2,B=(Ixx·sin2d+Iyy·cos2d)−m·h2,C=Izz,D=Ixy=Iyx=(Ixx−Iyy)cosd·sind−m·h2,

where *I*_*xx*_, *I*_*yy*_, and *I*_*zz*_ are the moments of inertia, with respect to the principal axes of the ellipsoid, defined as follows:

(2) Ixx=15m(b2+c2),Iyy=15m(a2+c2),Izz=15m(a2+b2),

(5) $$\begin{array}{c} E_{1}=\left|\begin{array}{ccc} Q_{1} & D-m\cdot x_{1}\cdot x_{2} & m\cdot \left(h-x_{3}\right)\cdot x_{1}\\ Q_{2} & B+m\left[x_{1}^{2}+{\left(h-x_{3}\right)}^{2}\right] & m\cdot \left(h-x_{2}\right)\cdot x_{1}\\ Q_{3} & m\cdot \left(h-x_{2}\right)\cdot x_{1} & C+m\cdot \left(x_{1}^{2}+x_{2}^{2}\right) \end{array}\right|, \end{array}$$

(6) $$\begin{array}{c} E_{2}=\left|\begin{array}{ccc} A+m\left[x_{2}^{2}+{\left(h-x_{3}\right)}^{2}\right] & Q_{1} & m\cdot \left(h-x_{3}\right)\cdot x_{1}\\ D-m\cdot x_{1}\cdot x_{2} & Q_{2} & m\cdot \left(h-x_{2}\right)\cdot x_{1}\\ m\cdot \left(h-x_{3}\right)\cdot x_{1} & Q_{3} & C+m\cdot \left(x_{1}^{2}+x_{2}^{2}\right) \end{array}\right|, \end{array}$$

(7) $$\begin{array}{c} G=\left|\begin{array}{ccc} A+m\left[x_{2}^{2}+{\left(h-x_{3}\right)}^{2}\right] & D-m\cdot x_{1}\cdot x_{2} & Q_{1}\\ D-m\cdot x_{1}\cdot x_{2} & B+m\left[x_{1}^{2}+{\left(h-x_{3}\right)}^{2}\right] & Q_{2}\\ m\cdot \left(h-x_{3}\right)\cdot x_{1} & m\cdot \left(h-x_{2}\right)\cdot x_{1} & Q_{3} \end{array}\right|. \end{array}$$

*Q*_*k*_(*k* = 1, 2, 3) that appear in determinants *E*1, *E*2, *E*3 are defined by (8):

(8) $$\begin{array}{c} Q_{k}=F_{k}+R_{k}+S_{k}, \end{array}$$

with *F*_*k*_, *R*_*k*_, *S*_*k*_(*k* = 1,2, 3):

(9) F1=T1+mg·[(x3−h)·μ2−x2·μ3],F2=T2+mg·[(h−x3)·μ1+x1·μ3],F3=T3+mg·[(x2·μ1−x1·μ2)],R1=[D·ω1+(B−C)·ω2]·ω3,R2=[(C−A)·ω1−D·ω2]·ω3,R3=D·(ω22−ω12)+(A−B)·ω1·ω2,S1=m·[(h−x3)·ζ2+x3·ζ3],S2=m·[(x3−h)·ζ1−x1·ζ3],S3=m·[x1·ζ2−x2·ζ1],

where *T*_*k*_ = −*σ* · *ω*_*k*_(*k* = 1,2, 3) is the action of a torque with a positive constant standing for the air resistance coefficient. The underlying idea is that air resistance may be the principal energy-dissipating mechanism that must be taken into account. The rest of the parameters, appearing in equation set (9), are defined in the following set of (10):

(10) μ1=−cosα·sinβ,μ2=sinα,μ3=cosα·cosβ,ζ1=ω2·(v3−dx3dt)−ω3·(v2−dx2dt),ζ2=ω3·(v1−dx1dt)−ω1·(v3−dx3dt),ζ3=ω1·(v2−dx2dt)−ω2·(v1−dx1dt),

with *v*_*k*_ the coordinates of the ellipsoid's linear velocity:

(11) v1=ω2·(h−x3)+ω3·x2,v2=−ω3·x1−ω1·(h−x3),v3=−ω1·x2+ω2·x1.

Parameters *a*, *b*, *c* are the ellipsoid's dimensions on its axes, while *z* is the distance of the top horizontal surface from its initial center of gravity. Parameter *M* is the ellipsoid's mass. Variable *δ* (the angle between the vertical axis of the ellipsoid and the flat surface) is defined by (12)

(12) $$\begin{array}{c} \delta =\cos^{-1}\mu_{3}. \end{array}$$

**Figure 2:**
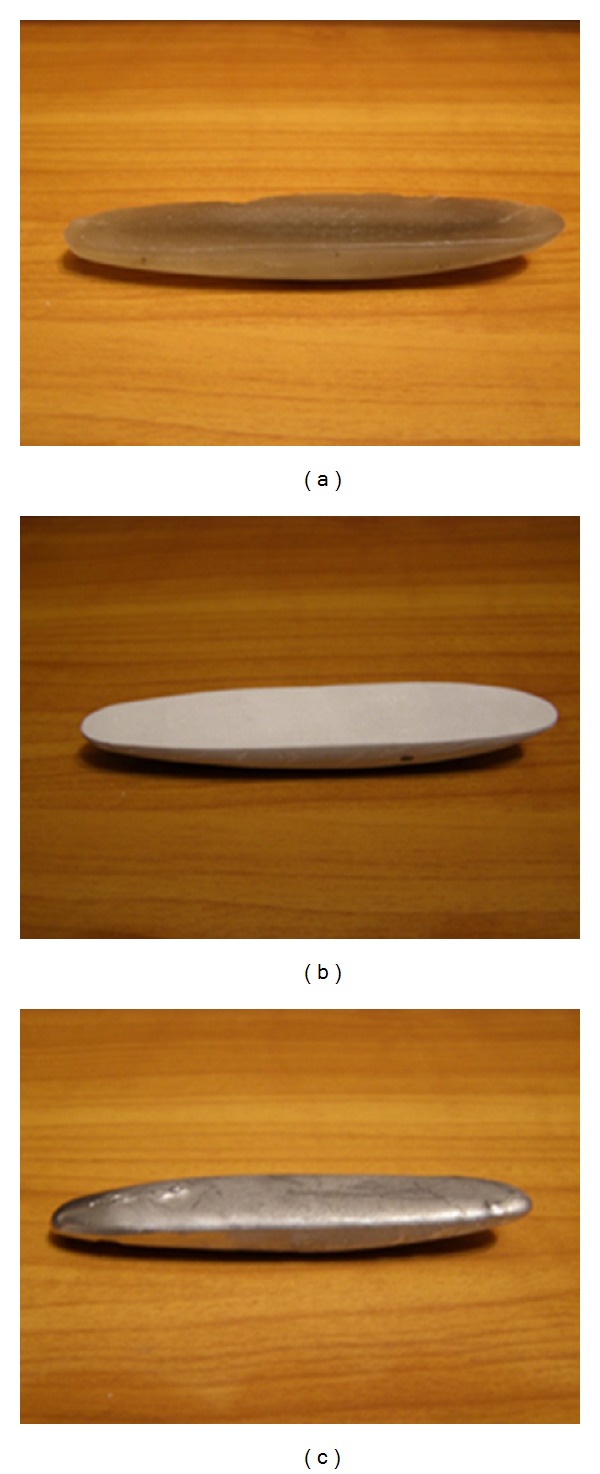
The three rattleback prototypes made of (a) wax, (b) gypsum, and (c) lead-solder.

In the work presented in this paper, three rattlebacks made of three different materials were constructed. The materials used were wax, gypsum, and lead-solder and the prototypes appear in Figure 2. All three bodies were tested on whether they behaved as expected, and moreover, the total time until they stopped, before reversing their spin direction, was measured and it was found to be the same with the time provided by Kane's set (1) of equations, thus confirming both the realistic value of Kane's mathematical approach and the constructed objects' right behaviour.

In order to experimentally define parameters *a*, *b*, *c*, *h*, and *M*, appearing in Kane's model, all three rattlebacks had their dimensions measured and were weighted. As a result, the following parameter sets have emerged and they are presented in Table 1.

Then, the corresponding mass moments of inertia were calculated for each ellipsoid and they possessed the values appearing in Table 2. It is noted that the three ellipsoids were constructed having their dimensions almost the same (see Table 1), in an effort to be comparable one to the other. Consequently, only masses possessed different values, resulting of course in different mass moments of inertia.

Simulation of the experimental defined mathematical model, proposed by Kane and Levinson [4] was run, with the following initial conditions: *α* = 0.5 degrees, *β* = 0.5 degrees, *γ* = 0 degrees, *ω*_1_ = *ω*_2_ = 0, *ω*_3_ = −5 rad/s, *σ* = 0 (no air-resistance).

## 3. Time-Series Analysis

In order to explore and study rattleback's dynamics when it is initially gyrated to the counter-clockwise direction, the set of the six nonlinear equations (1), modeling (according to Kane) rattleback's dynamical behaviour, was numerically solved with Matlab's ODE45. This procedure was executed for the three cases of rattleback's different material realizations, as these are mentioned in Table 1.

**Figure 3:**
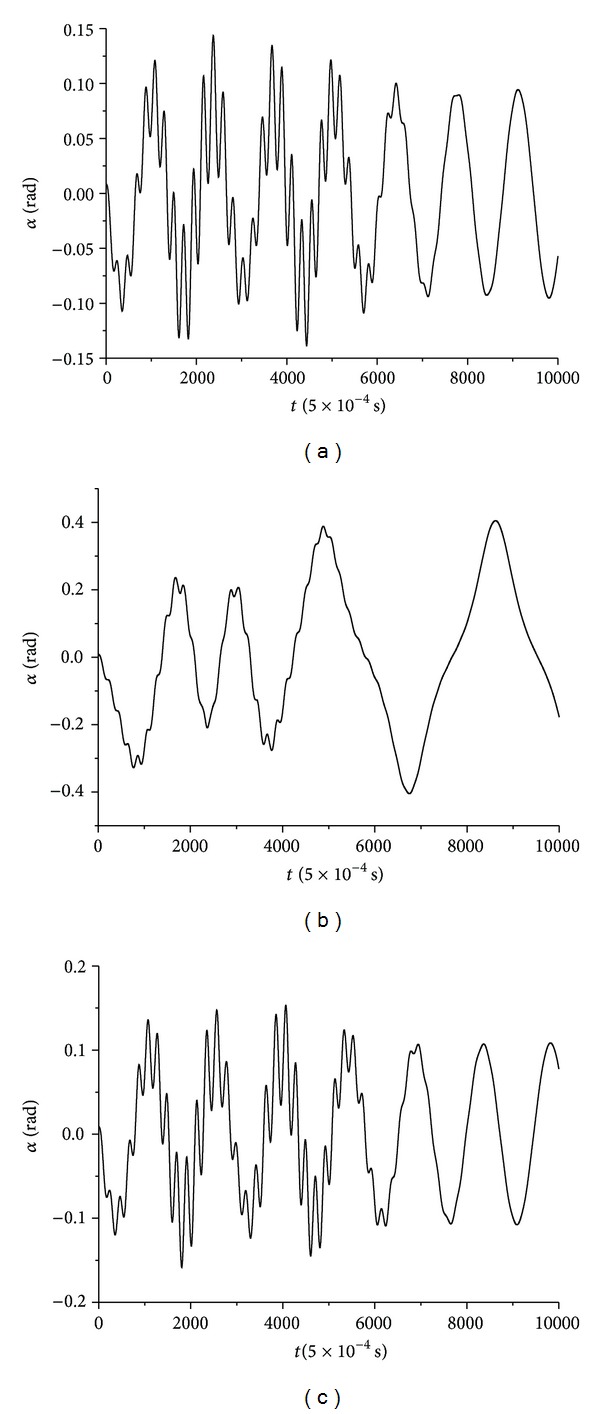
Roll angle *α* time-series for rattlebacks made of (a) wax (b) gypsum, and (c) lead-solder.

**Figure 4:**
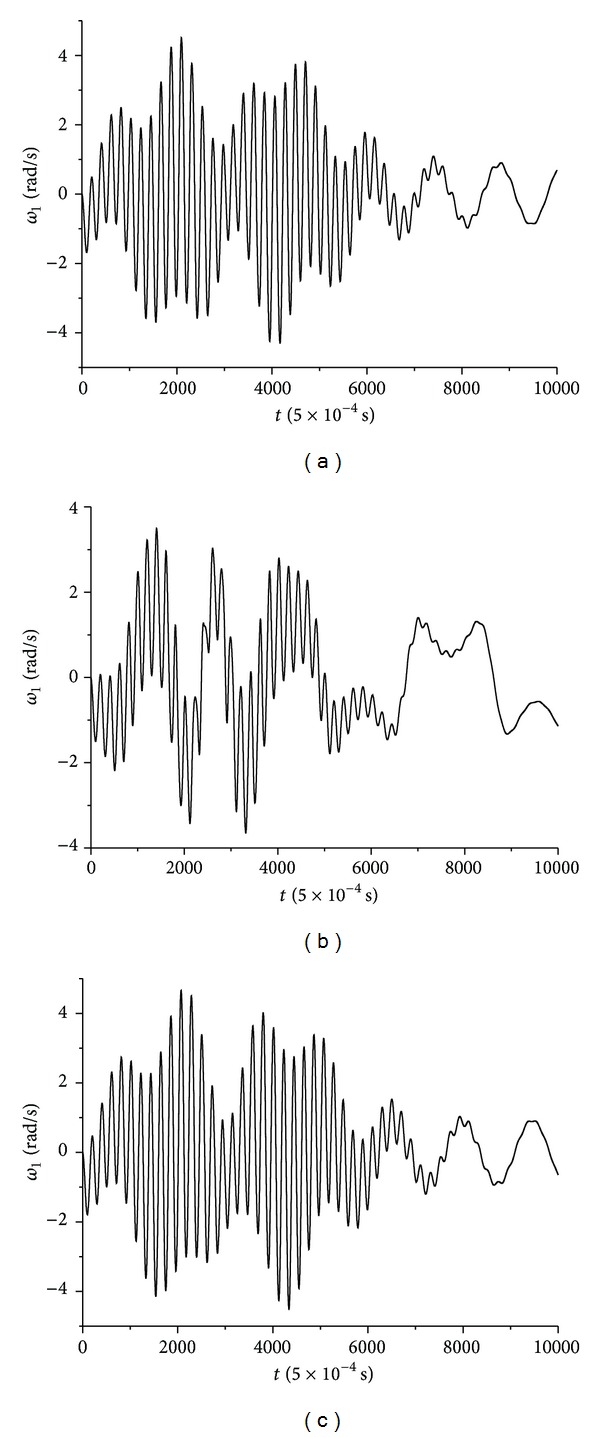
Ellipsoid spin rate *ω*_1_ time-series in the case of (a) wax, (b) gypsum, and (c) lead solder.

**Figure 5:**
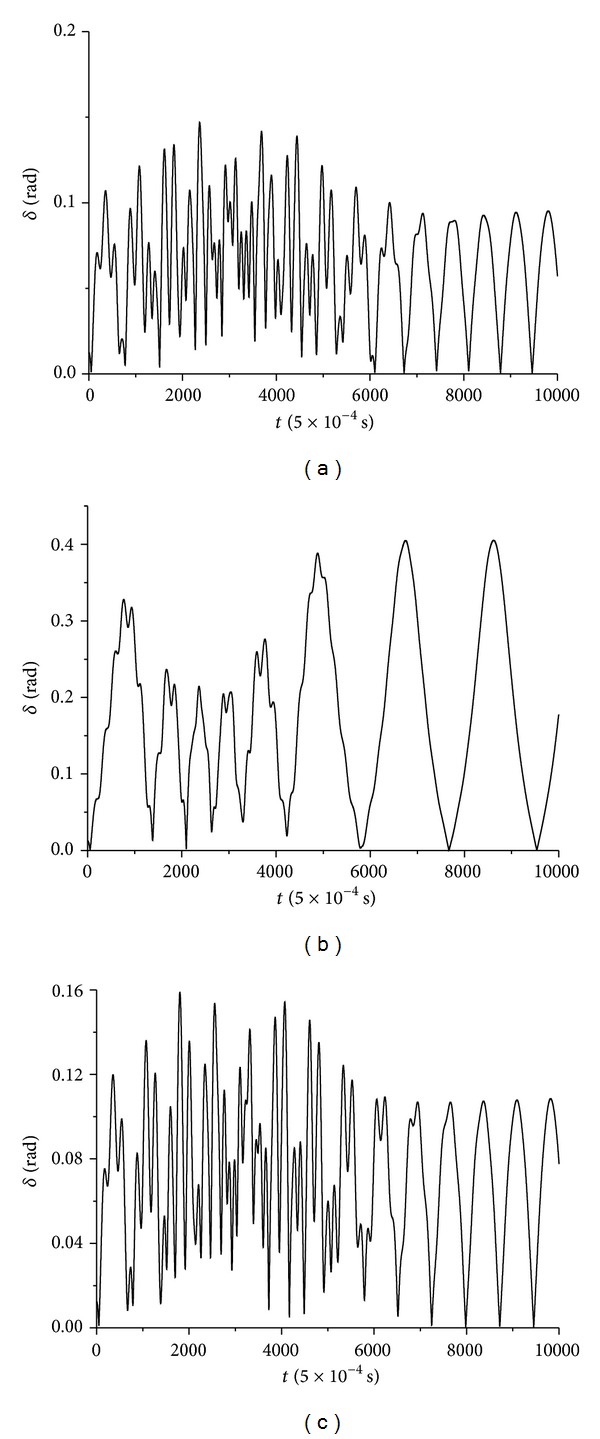
Ellipsoid's angle *δ* between the vertical axes of the ellipsoid and its surface time-series in the case of (a) wax (b) gypsum, and (c) lead solder.

As already described in this case, rattleback's motion stops and oscillations take place until it starts to rotate in the clockwise direction. During the oscillation time period, the variable time-series demonstrating an irregular behaviour, hinting to a deterministic chaotic one, in all three cases were roll angle *α*, the corresponding spin rate *ω*_1_, and the angle between *δ* the ellipsoid's vertical axis and the plain surface on top. These time-series were calculated for the three rattleback realizations. In particular the differential equations of set (1) were solved (numerically) with a step *h* = 0.0005, registering *N* = 10000 points and the solutions are presented in Figures 3, 4, and 5. In these figures the evolvement of the selected variables appears for the time period from the beginning of the oscillations to a little before the beginning of the clockwise rotation.

In Figure 3 the roll angle *α*(*t*) time-series for wax, gypsum, and lead solder made rattlebacks are presented, as these were numerically calculated.

From the same numerical calculations the time-series of spin rate *ω*_1_(*t*) for wax, gypsum, and lead solder are presented in Figure 4.

Finally, in Figure 5 the angle *δ*(*t*) between the vertical axes of the ellipsoid and the flat surface *δ* time series for the cases of wax, gypsum, and lead solder materials appears.

From all these three figures, it is apparent that an irregular behaviour emerges when rattleback begins to oscillate during the transition from a counterclockwise to a clockwise direction. This irregular oscillation takes place for a while and it is degraded to an almost periodic one until it starts rotating again.

## 4. Evaluation

In this section nonlinear analysis and evaluation of the calculated irregular oscillations of the three variables appearing in Figures 3, 4, and 5 for the three discrete cases of different material-made rattlebacks are presented. Consequently, the calculated time-series *α* = *α*(*t*), *ω*_1_ = *ω*(*t*), and *δ* = *δ*(*t*) for wax (Figure 3), gypsum (Figure 4), and lead solder (Figure 5) materials were studied by applying well-known Grassberger-Procaccia method [10].

As a first step, utilizing Takens theory [11], a topologically equivalent to the original phase space was reconstructed for each of the three calculated time-series. In order to achieve this correlation, integral *C*(*r*) was calculated from the time-series appearing in Figures 3–5. Correlation integral is generally defined as [11]

(13) C(r)=1Npairs∑l=1,j=l+wNH(r−||X→l−X→j||),

where *N* is the number of the corresponding data points, *W* is the Theiler window [10], *H* is the Heaviside function, and *N*_pairs_ is defined by the following relation:

(14) $$\begin{array}{c} N_{\text{pairs}}=\frac{2}{\left(N-m+1\right)\left(N-m+W+1\right)}, \end{array}$$

with *m* being the embedding dimension.

It is apparent that the summation in (13) counts the number of pairs for which the distance, that is, the Euclidean norm, is less than *r* in an *m* dimensional Euclidean space. In this case, the number of the experimental data points was *N* = 10000. Considering the *m* dimensional space, each vector should be given by

(15) $$\begin{array}{c} \vec{X}=\left\{\delta \left(t\right),\delta \left(t+\tau \right),\delta \left(t+2\tau \right),\ldots ,\delta \left[t+\left(m-1\right)\tau \right]\right\}, \end{array}$$

and it would represent a point in the *m* dimensional phase space [10]. In (15), *τ* stands for the delay time determined by the first minimum of mutual information function *I*(*τ*).

As Theiler pointed out, if strongly correlated points are not to be neglected, a spuriously low dimension estimate may be obtained. Consequently, a correction by introducing parameter *W* (the Theiler window) should be introduced. However, since there is no standard method for choosing *W*, this may be determined by absolute minimum of mutual information [12]. Hence, we can use these values for phase space reconstruction. With (13) dividing the considered *m* dimensional phase space into hypercubes with a linear dimension *r*, all points with mutual distances less than *r* are counted. Then, if the attractor is a strange one, the correlation integral will be proportional to *r* ^*ν*^, where *v* is a measure of the attractor's dimension called correlation dimension.

The above described method has been applied to the timeseries' of Figures 3–5.

### 4.1. Roll Angle *α*(*t*) Time-Series Chaotic Evaluation

**Figure 6:**
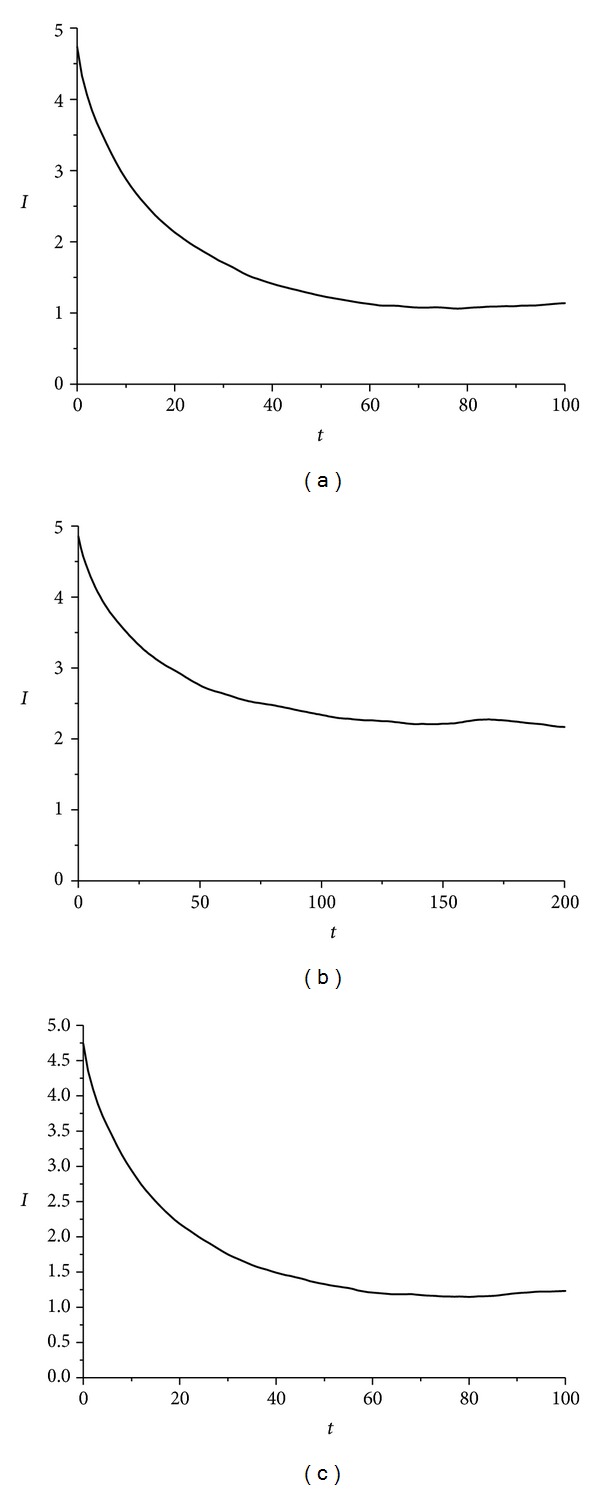
Average mutual information *I*(*τ*) versus *τ* time delay for roll angle *α*(*t*) time-series, in the case of (a) wax, *τ* = 63, *W* = 78, (b) gypsum, *τ* = 140, *W* = 200, and (c) lead-solder, *τ* = 65, *W* = 80. *τ* is the first minimum and *W* is the absolute minimum and this value is regarded as the Theiler window.

In this subsection three different material made rattlebacks' dynamics are evaluated by studying the roll angle *α*(*t*) time-series. As shown in Figure 6(a), for wax the ellipsoid's mutual information exhibits a local minimum at *τ* = 63 steps and an absolute minimum at *W* = 78 steps. Thus, this value (*τ* = 63) should be considered as the optimum delay time while the Theiler window is *W* = 78. The same way, for the gypsum ellipsoid, mutual information (appears in Figure 6(b)) exhibits a local minimum at *τ* = 140 steps (optimum delay time) and an absolute minimum at *W* = 200 steps (Theiler window value). Finally, in the case of the lead solder ellipsoid mutual information (appears in Figure 6(c)) exhibits a local minimum at *τ* = 65 steps (optimum delay time) and an absolute minimum at *W* = 80 steps (Theiler window value).

**Figure 7:**
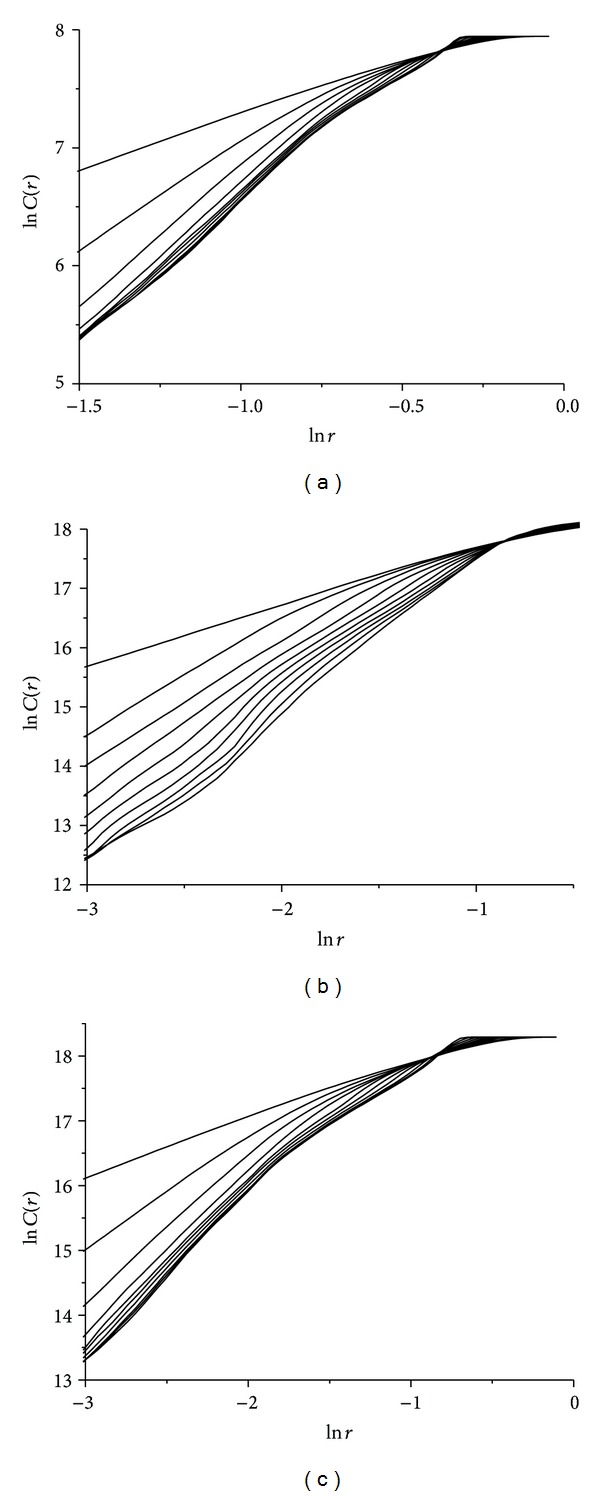
Relation between ln*C*(*r*) and ln*r* for different embedding dimensions *m* for roll angle *α* time-series for (a) wax, (b)gypsum, and (c) lead-solder, materials.

By using the optimum delay time calculated above, scaling of correlation integral *C*(*r*) according to *r*, for different embedding dimensions *m*, is presented in Figure 7. These double logarithmic plots illustrate the relationship between ln*C*(*r*) and ln*r* in all three cases of wax (Figure 7(a)), gypsum (Figure 7(b)), and lead solder (Figure 7(c)) rattlebacks.

**Figure 8:**
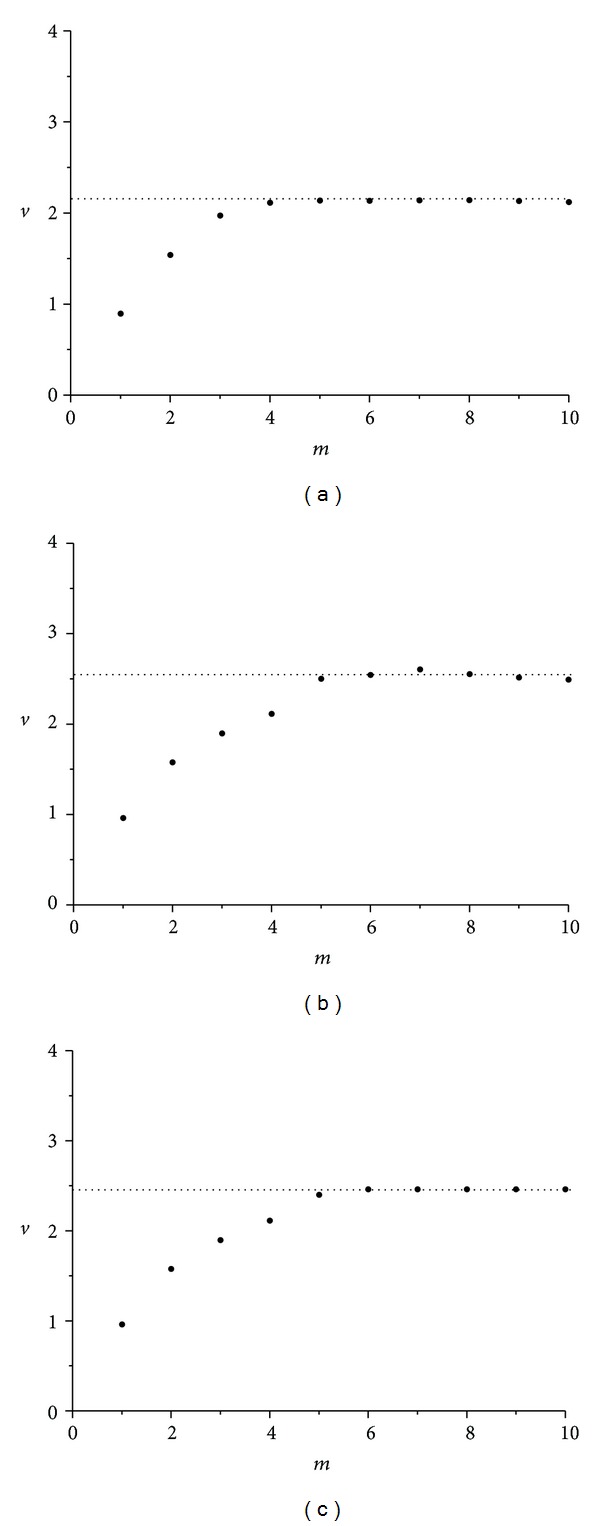
Correlation dimension *v* versus *m* embedding dimension for roll angle *α*(*t*) time-series in the cases of (a) wax, *v* = 2.13, (b) gypsum, *v* = 2.54, and (c) lead solder, *v* = 2.06. In all cases, minimum embedding dimension under these conditions is 3.

The corresponding average slopes *v* (correlation dimensions) of the linear parts of the three diagrams of Figure 7 as a function of the embedding dimension *m* appear in Figure 8. As seen in the corresponding figures, for higher values of embedding dimension *m*, slopes *v* (correlation dimension) tend to saturate to the noninteger value of *v* = 2.13 (for wax), *v* = 2.54 (for gypsum), and *v* = 2.06 (for lead solder), providing a confirmation of time-series *α*(*t*) chaotic nature, in all three cases [10, 12].

According to Abarbanel [13], the closest integer above the correlation dimension provides with the proper minimum embedding dimension *m*_min_, which in this case possesses the value *m*_min_ = 3 for all three materials. This minimum embedding dimension is referred to the system's attractor under the specific conditions and it reveals the essential dimension of the corresponding dynamical system phase space (and the number of the essential variables), necessary to model the dynamics of the attractor.

On the other hand, the sufficient phase space dimension, necessary to fully describe the global dynamics of the system, can also be experimentally identified in Figure 8, by identifying the embedding dimension where the correlation exponent reaches its saturation value [14]. In this case, it is apparent that this happens after the 6th embedding dimension. Thus, the sufficient phase space embedding dimension for the attractor, describing rattleback's global dynamics, is equal to 6, as confirmed by the number of state variables.

### 4.2. Spin Rate *ω*_1_(*t*) Time-Series Chaotic Evaluation

In this subsection rattlebacks' dynamics are evaluated by studying the spin rate *ω*_1_(*t*) time-series. Again this evaluation takes place for the three different material-made rattlebacks.

**Figure 9:**
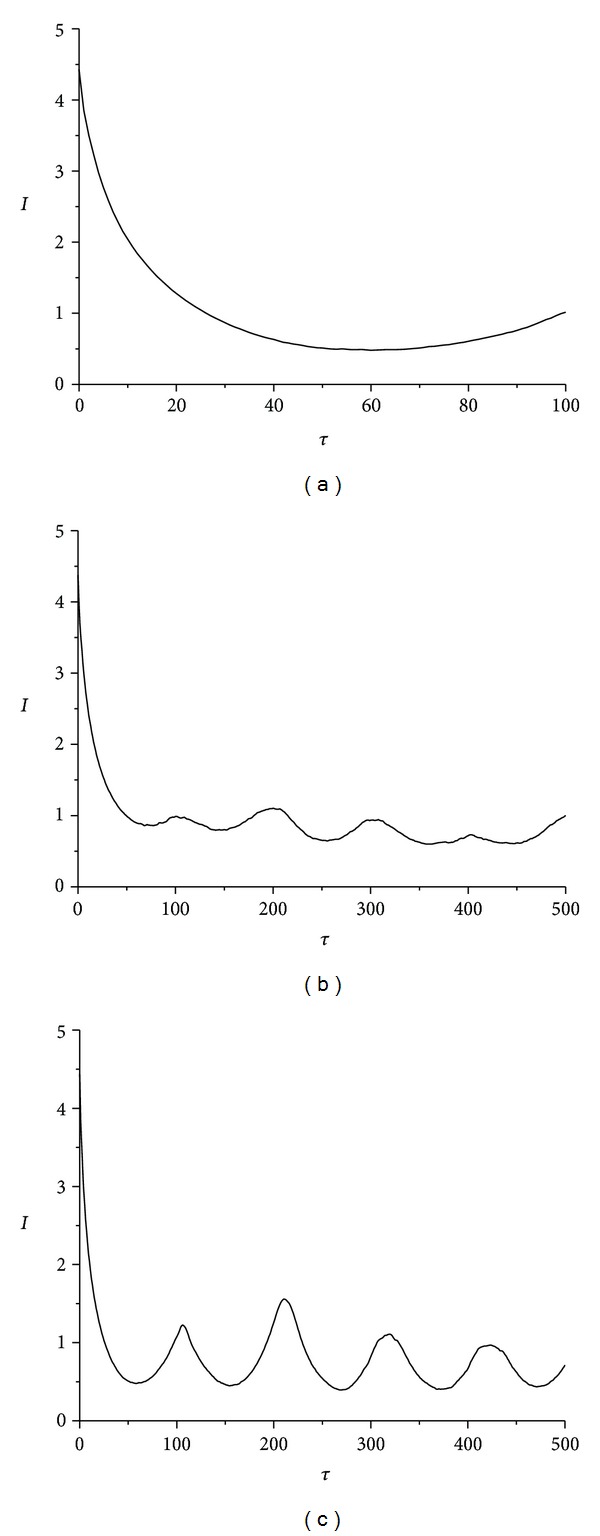
Average mutual information *I*(*τ*) versus *τ* time delay for spin rate *ω*_1_(*t*) time-series in the case of (a) wax, *τ* = 53, *W* = 60, (b) gypsum, *τ* = 63, *W* = 358, and (c) lead solder, *τ* = 58, *W* = 58. *τ* is the first minimum and *W* is the absolute minimum and this value is regarded as the Theiler window.

In Figure 9, the essential data needed for further evaluating rattleback's chaotic dynamics, by utilizing mutual information *I*(*t*), is extracted. Again the first local minimum determines the delay time and the absolute minimum Theiler window.

In Figure 9(a), in the case of wax-made ellipsoid, mutual information exhibits a local minimum at *τ* = 53 steps and an absolute minimum at *W* = 60 steps. The same way, for the gypsum-made ellipsoid, mutual information (appears in Figure 9(b)) exhibits a local minimum at *τ* = 63 steps (optimum delay time) and an absolute minimum at *W* = 358 steps (Theiler window value). Finally, in the case of the lead solder ellipsoid, mutual information (appears in Figure 9(c)) exhibits a local minimum at *τ* = 58 steps (optimum delay time) and an absolute minimum at *W* = 58 steps (Theiler window value).

**Figure 10:**
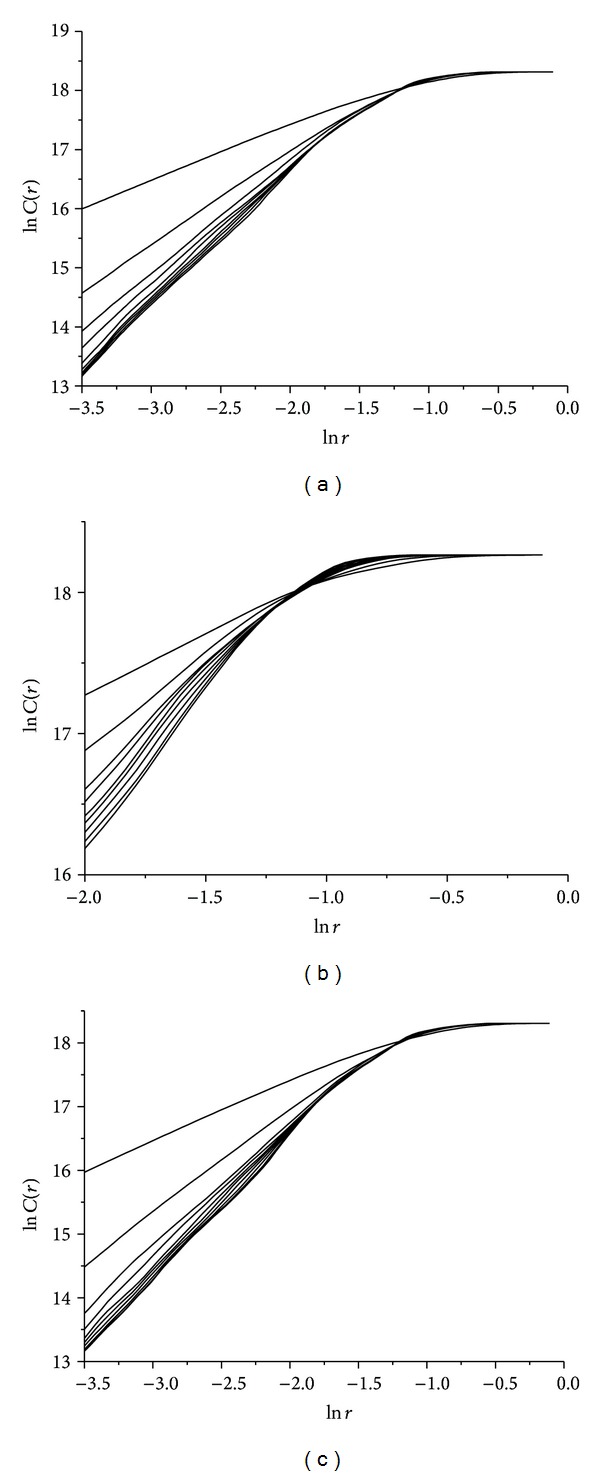
Relation between ln*C*(*r*) and ln*r* for different embedding dimensions *m* for spin rate *ω*_1_(*t*) time-series, for (a) wax, (b) gypsum, and (c) lead solder.

By using the optimum delay time calculated above, scaling of correlation integral *C*(*r*) according to *r*, for different embedding dimensions *m*, is presented in Figure 10. These double logarithmic plots illustrate the relation between ln*C*(*r*) and ln*r* in all three cases of wax (Figure 10(a)), gypsum (Figure 10(b)), and lead solder (Figure 10(c)) rattlebacks.

**Figure 11:**
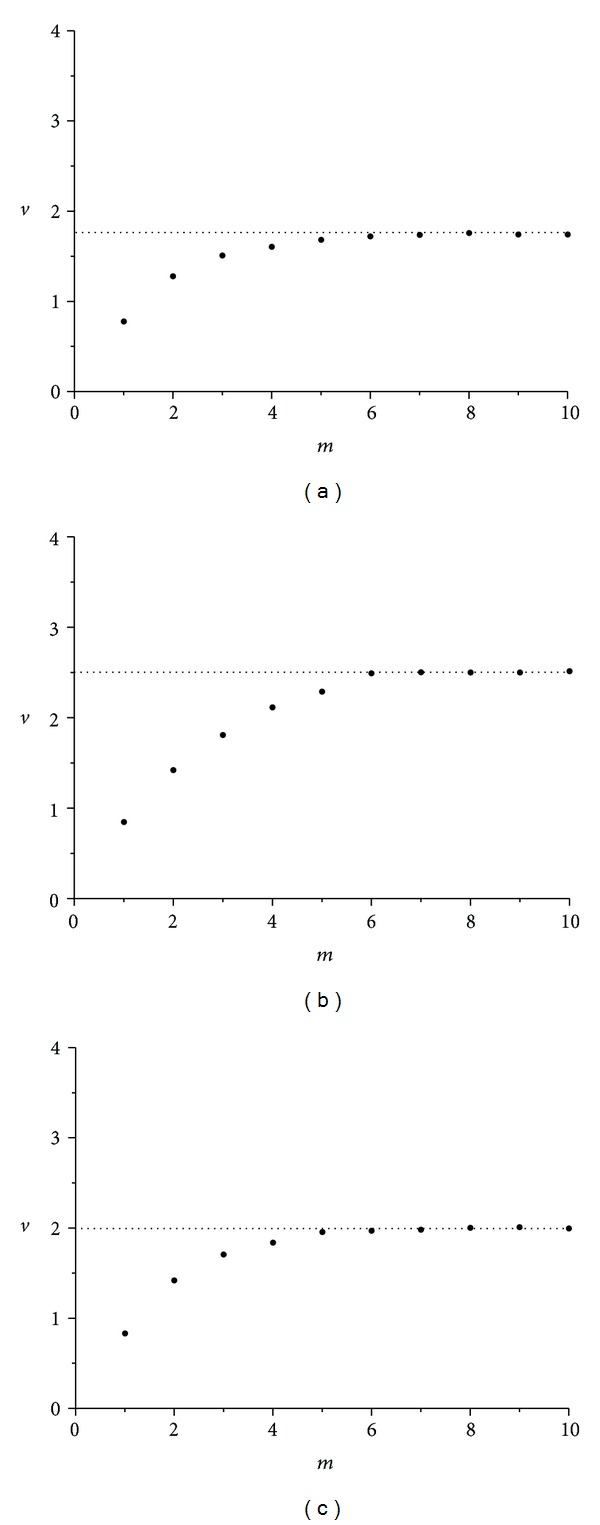
Correlation dimension *v* versus *m* embedding dimension for spin rate *ω*_1_(*t*) time-series in the cases of (a) wax, *v* = 1.71 (b) gypsum, *v* = 2.49, and (c) lead solder, *v* = 1.97. Minimum embedding dimension is not the same in all cases, revealing the not so common dynamics of rattleback.

Again the average slopes *v* of the linear parts of the three diagrams, appearing in Figure 10, as a function of embedding dimension *m* (corresponding to correlation dimensions), are formatting the diagrams in Figure 11. In these diagrams, for higher values of embedding dimension *m*, slopes tend to saturate to the noninteger values of *v* = 1.71 (for wax), *v* = 2.49 (for gypsum), and *v* = 1.97 (for lead solder), thus providing again a confirmation of this time-series *ω*_1_(*t*) chaotic nature, in all three cases [10, 12].

Taking into account [13], the minimum embedding dimension is once more determined by the closest integer above the correlation dimension. In this case, this provides the proper minimum embedding dimension *m*_min_, which in this case possesses the value *m*_min_ = 2 for wax and lead solder, while it is *m*_min_ = 2 for gypsum. It is noted again that this minimum embedding dimension is referred to the system's attractor under the specific conditions and it reveals the essential dimension of the corresponding dynamical system phase space (and the number of the essential variables) necessary to model the dynamics of the attractor, once more revealing the very interesting and not so common dynamics exhibited by rattleback.

Again, the sufficient phase space dimension, necessary to fully describe the global dynamics of the system, possesses the value 6 as expected.

### 4.3. Angle *δ*(*t*) Time-Series Analysis

**Figure 12:**
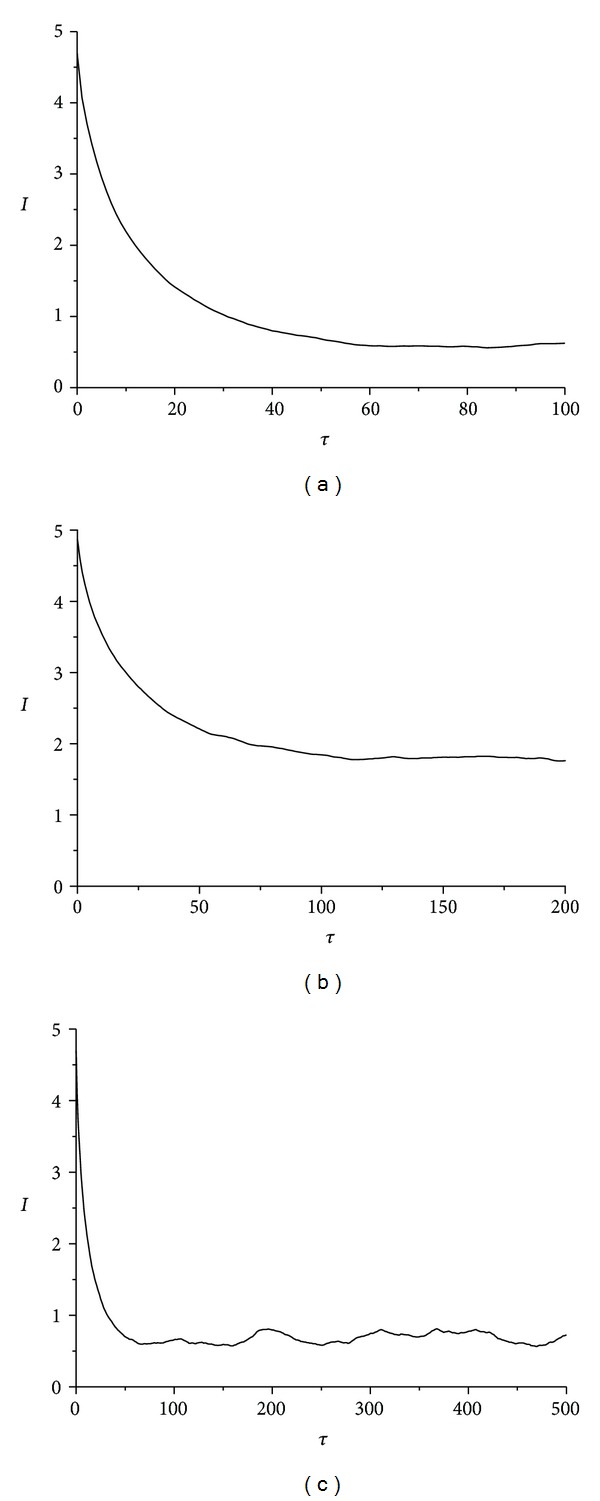
Average mutual information *I*(*τ*) versus *τ* time delay for angle *δ*(*t*) time-series in the case of (a) wax, *τ* = 61, *W* = 84, (b) gypsum, *τ* = 114, *W* = 197, (c) lead solder, *τ* = 55, *W* = 470. It is noted that *τ* determines the proper delay time and *W* the Theiler window.

The last time-series, evaluated for producing the essential metrics regarding rattleback's chaotic behaviour, is that of the angle *δ*(*t*) between the vertical axis of the ellipsoid and the flat surface. This variable is defined by (12) in the second section and it is produced by two state variables *α*(*t*) and *β*(*t*). Like the previous two cases, calculation of mutual information *I*(*t*) provided the necessary elements to further evaluate the chaotic dynamics demonstrated, according to [10, 11]. The first local minimum determines the delay time and the absolute minimum (Theiler window). So as shown in Figure 12(a) and in the case of wax-made ellipsoid, mutual information exhibits a local minimum at *τ* = 61 steps and an absolute minimum at *W* = 84 steps. The same way, for the gypsum-made ellipsoid, mutual information (appears in Figure 12(b)) exhibits a local minimum at *τ* = 114 steps (optimum delay time) and an absolute minimum at *W* = 197 steps (Theiler window value). Finally, in the case of the lead solder ellipsoid, mutual information (appears in Figure 12(c)) exhibits a local minimum at *τ* = 55 steps (optimum delay time) and an absolute minimum at *W* = 470 steps (Theiler window value).

**Figure 13:**
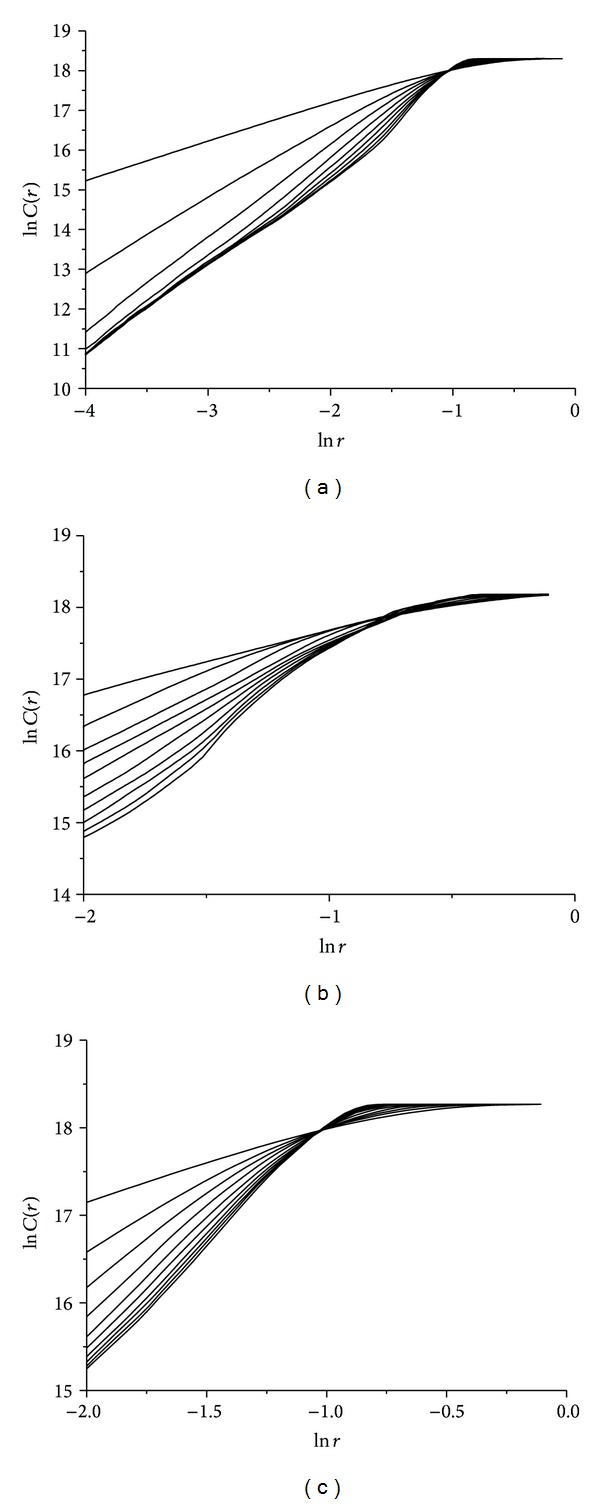
Relation between ln*C*(*r*) and ln*r* for different embedding dimensions *m* for angle *δ*(*t*) time-series in the cases of rattlebacks made of (a) wax, (b) gypsum, and (c) lead solder, materials.

By using the optimum delay time calculated above, scaling of correlation integral *C*(*r*) according to *r*, for different embedding dimensions *m*, is presented in Figure 13 (for all three cases of wax —Figure 13(a)— gypsum —Figure 13(b)— and lead solder —Figure 13(c)).

**Figure 14:**
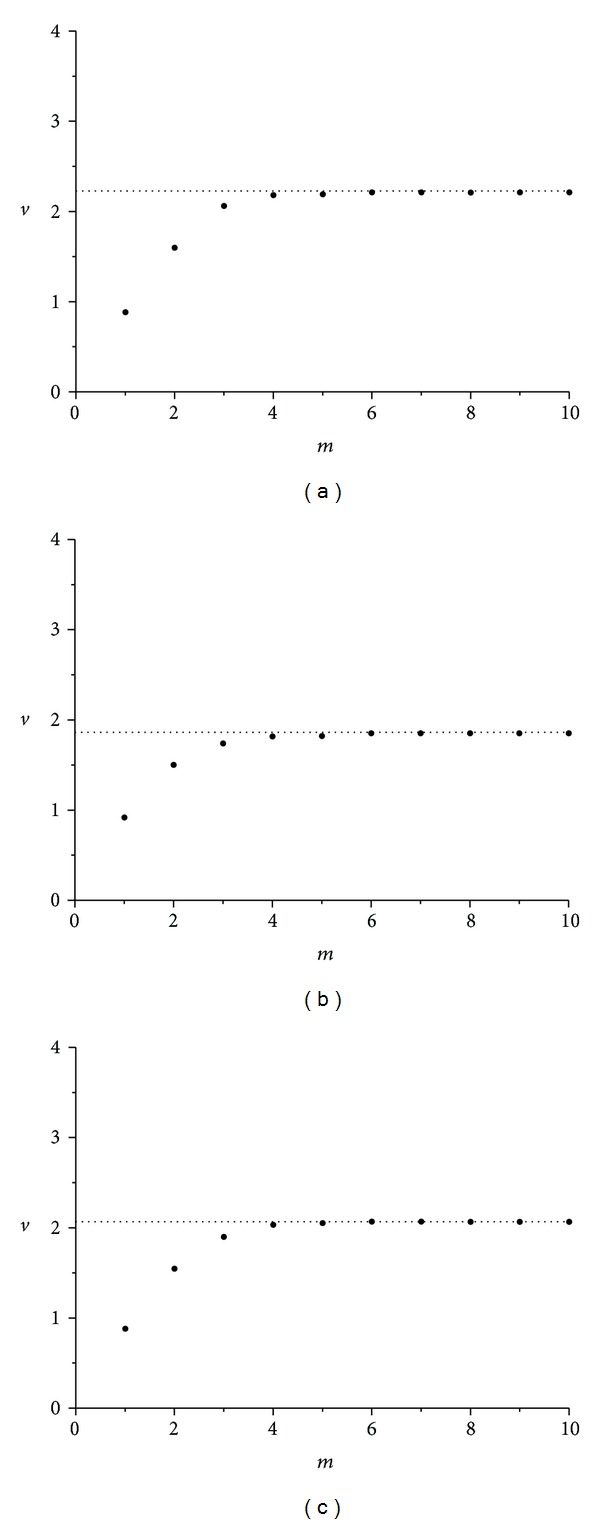
Correlation dimension *v* versus *m* embedding dimension for angle *δ*(*t*) time-series in the cases of (a) wax, *v* = 2.20, (b) gypsum, *v* = 1.85, and (c) lead solder, *v* = 2.06. Minimum embedding dimension is not the same in all cases, revealing the not so common dynamics of rattleback.

As already described, the average slopes *v* of the linear parts of the three diagrams, appearing in Figure 14, as a function of embedding dimension *m* (corresponding to correlation dimensions), are formatting the diagrams in Figure 14. In these diagrams, the correlation dimension appears to possess noninteger values: *v* = 2.20 for wax, *v* = 1.85 for gypsum, and *v* = 2.06 for lead solder, thus providing again a confirmation of this time-series *ω*_1_(*t*) chaotic nature, in all three cases [10, 12]. Consequently, the minimum embedding dimensions according to [13] are *m*_min_ = 3 for wax and lead solder, while it is *m*_min_ = 2 for gypsum. Again, the sufficient phase space dimension, necessary to fully describe the global dynamics of the system, possesses the value 6, as expected by the theoretical model.

## 5. Conclusions

In this paper chaotic dynamics emerging during the oscillations, taking place in the procedure of rattleback's spin direction reversing, have been studied. This study utilized Kane's mathematical model. The model's parameter values were determined by measuring and weighting real rattleback prototypes made of three different materials: wax, gypsum, and lead solder.

The evaluation of the related results clearly hint at the demonstration of chaotic dynamics (during the process of reversing spin direction, in case rattleback is initially counterclockwise gyrated). However, there are differences in the strength of chaos demonstrated by different material-made prototypes, revealing rattleback's interesting and rather curious dynamics. In particular, studying the time-series of roll angle *α* correlation dimension in gypsum-made ellipsoid (2.54) was larger than in the case of wax (2.13), which was larger than in the case of lead-solder (2.06). The same behaviour appears for the spin rate *ω*_1_ time-series, where the corresponding strange attractor for wax, gypsum, and lead solder had correlation dimensions of 1.71, 2.49, 1.97, respectively. However, comparing the correlation dimensions for the time-series of angle *δ* between the vertical axes of the ellipsoid and the surface, it was found that the gypsum-made ellipsoid demonstrated the smallest (*v* = 1.85) value, compared to the wax-made (*v* = 2.20) and the lead-solder-made (*v* = 2.06).

These differences in the resulting values of correlation dimension could be attributed to the slightly different dimension of the prototypes and of course their masses. It is apparent that these small changes finally lead to a different chaotic movement (oscillations) in the sense of chaos strength. In any case it seems that the dominant role in the appearance of this chaotic behaviour belongs to the shape of the ellipsoid and in no case to the prototype's construction material.

## Figures and Tables

**Table 1:** Kane's model parameters for three rattlebacks made of different materials.

	*a* (m)	*b* (m)	*c* (m)	*h* = 3*c*/8 (m)	*M* (kgr)
Wax ellipsoid	0.0482	0.00965	0.0117	0.044	0.0106
Gypsum ellipsoid	0.04835	0.00925	0.0016 m	0.006	0.0154
Lead-solder ellipsoid	0.04835	0.00925	0.0016 m	0.006	0.0205

**Table 2:** Mass moments of inertia were calculated for each ellipsoid.

	*A* (Kgr·m^2^)	*B* (Kgr·m^2^)	*C* (Kgr·m^2^)	*D* (Kgr·m^2^)
Wax ellipsoid	0.0482	0.00965	0.0117	0.044
Gypsum ellipsoid	0.04835	0.00925	0.0016m	0.006
Lead-solder ellipsoid	0.04835	0.00925	0.0016m	0.006
